# Problem based learning approach increases the academic satisfaction of health science students in Ethiopian universities: a comparative cross sectional study

**DOI:** 10.1186/s12909-022-03397-5

**Published:** 2022-05-01

**Authors:** Selamawit Girma Tadesse, Dereje Girma Tadesse, Eyaya Habtie Dagnaw

**Affiliations:** 1 Department of Midwifery, College of Health Sciences, Debretabor University, Debre Tabor, Ethiopia; 2 Nutrition officer, World Health Organization, Bahirdar, Ethiopia

**Keywords:** Academic satisfaction, Institution based, Comparative cross sectional, Problem based learning, Lecture based learning

## Abstract

**Background:**

Problem based learning is being highly implemented in many medical schools worldwide due to its perceived advantages including improvement of problem-solving abilities, development of communication skills, creation and development of critical thinking skill, and making of individuals to be lifelong learners & responsible for their own learning process.

**Objective:**

The study aimed to compare academic satisfaction of Problem and Lecture based learning of regular undergraduate health science students in Ethiopian Universities, 2021.

**Method:**

Institution based comparative cross sectional study was conducted from February 1–30, 2021. Data were collected using a pretested structured and self-administered questionnaire among 850 eligible students from two Universities. Data were entered into EPI info version 6.04 and analyzed using SPSS version 23. Binary Logistic regression model was fitted to identify factors associated with academic satisfaction considering the association to be significant *p*- value < 0.05.

**Result:**

The study result revealed that the magnitude of academic satisfaction among problem based and lecture based learning students were 50.9 and 49.9% respectively. Similarly, problem based learning students were more likely to be academically satisfied than lecture based learning students in their type of curriculum with (AOR = 1.50, 95% CI = 1.02, 2.21). Experience of classroom distress (AOR = 1.93, 95% CI = 1.22, 3.06), quality of teaching (AOR = 0.54, 95% CI = 0.34, 0.86), relationship with classmates (AOR = 0.33, 95% CI = 0.13, 0.80), course content (AOR = 0.56, 95% CI = 0.33, 0.93) and accessibility of technology in the campus (AOR = 0.62, 95% CI = 0.40, 0.96) were the significant factors of academic satisfaction of problem based learning students. Year of study (AOR = 0.29, 95% CI = 0.17, 0.48), quality of teaching (AOR = 0.51, 95% CI = 0.31, 0.85), course content (AOR = 0.59, 95% CI = 0.35, 0.97) and energy & effort (AOR = 0.55, 95% CI = 0.35, 0.88) were significantly associated with academic satisfaction among lecture based learning students.

**Conclusion:**

The study revealed that the academic satisfaction among problem based learning students was higher than lecture based learning students. Incorporating and implementing problem based learning as a formal instructional method in across the universities curriculum is recommended.

## Introduction

In health science education teaching and learning has become very important [1]. Problem-Based Learning (PBL) has been defined as an instructional method which uses well-constructed clinical problems as a context for students to learn problem-solving skills and acquire knowledge about the basic and clinical science [2]. It involves students working in small groups of five to ten to analyze problem or case scenarios as a basis for achieving the three learning domains [3]. There was a growing interest over the past decade for a paradigm shift from a traditional lecture-based mode of delivery to an active student-centered method [4]. PBL was first introduced in 1969 at McMaster University’s Faculty of Health Sciences in Canada [5].

Problem based learning is being highly implemented in many medical schools worldwide due to its perceived advantages including improvement of problem-solving abilities, increased knowledge retention, better integration of basic science and clinical skills, development of communication skills, creation and development of critical thinking skill, and making of individuals to be lifelong learners & responsible for their own learning process [6]. Globally, it is practiced in many health science faculties and currently, it serves as an active pedagogy in many disciplines to improve the teaching and learning processes in educational systems [3, 7].

Problem based learning models can be seen as pure type first introduced at McMaster University to the Hybrid models adopted in many other institutions including Ethiopia. The Hybrid models of PBL contain the skeleton of PBL but, were supported by different teaching activities like lecture and skill training sessions to provide the continual knowledge base [8].

Currently, active student involvement in the learning process is increasingly believed as one of the key indicators of quality education. Ethiopian Education and Training policy also promotes the implementation of active learning in all educational institutions including Universities [9]. Related to this PBL have been implementing in Debretabor University under Hybrid innovative curriculum since 2014 for Health science college students only and a year back the Ethiopian ministry of education in collaboration with the Ministry of Health was planned for its implementation in all higher education institutions as part of experimentation for health science faculty students whereas Mekelle University has been implementing the conventional lecture-based method to its Health science college students till the last 1 year starting from that it is on the way to start using PBL as one instructional method.

In line with education, student satisfaction defined as the favorability of a student’s subjective judgment of the various results and experiences associated with education [10].

Student satisfaction is basic for educational institutes, especially Universities but in reality, student satisfaction has never given an attention by academic authorities nor regarded as a matter of survival by higher education institutions. It implies that the impact of educational services given by a university on its student’s level of satisfaction has largely been an area that remains unexplored [11].

Therefore, this study is aimed at comparing student’s academic satisfaction with different instructional methods as it is one indicator of quality education which is among the three pillars of excellence or mission of higher education institutions shared in common. In addition, factors associated with academic satisfaction of students were also identified. Since there is no study conducted in the two study areas the result of this study will contribute as part of improving the satisfaction of regular undergraduate students in academic activities.

## Methods

A cross-sectional study was conducted at Debretabor and Mekelle Universities from February 1 to 30, 2021. Debretabor University is found in Debretabor town, Amhara regional state and have been use PBL as a formal educational method since 2014 for its health science students where as Mekelle University is found in Mekelle city, the capital of Tigray regional state, Ethiopia and was using LBL method. Both universities had a health science college with 3301 regular undergraduate students enrolled and six departments each were used for the study. Those departments were Medicine, Pharmacy, Midwifery, Nurse, Clinical laboratory and Anaesthesia. The total number of second and third year student’s for the academic year in both Universities were 1053.

### Study population

All second and third year regular undergraduate health Science College students found in Debretabor and Mekelle Universities during period of data collection. Inclusion criteria; All second and third year regular undergraduate health Science College students found in Debretabor and Mekelle Universities.

### Exclusion criteria

Students who transferred to DTU in the academic year from other universities those were not yet use PBL as one instructional method and who transferred to MU from other Universities that were using PBL as one instructional method were excluded. Since, there were no transferred students in both Universities all second and third year students (1053) were included in the study.

### Variables of the study

Academic satisfaction (five-point Likert scale merged as Satisfied or Unsatisfied) is the dependent variable. Independent variables were Socio-demographic characteristics: (Age, sex, religion, ethnic group, department, year of study & family’s educational level). Personal related factors: (Preferred learning style, Experience of classroom distress, GPA, Learning approach, Time investment, Energy and effort). Institutional factors: (Curriculum, teaching method, Quality of instructors, Quality of feedback, Student assessment, Staff helpfulness, Student centeredness) [12–24].

### Data collection procedure

Data were collected by self-administered structured and pretested questionnaire. The questionnaire was adapted from previous similar studies done at the College of Medicine, King Saud University, at King Abdulaziz University in Jeddah, Saudi Arabia and others [1, 25–41].

After the data has checked for completeness and accuracy, it was coded manually and then entered into Epi Info version 6.04 and exported to SPSS version 23 for analysis. Descriptive statistics were performed on numerical value, mean, frequencies to describe the study population about dependent and independent variables. The binary logistic regression model was fitted to determine the crude associations of each independent variable with academic satisfaction. Variables with a *p*-value of less than 0.05 were entered to multiple logistic regression for further analysis to control for confounding variables. Adjusted OR with 95% CI was used to estimate the strength of the association between independent variables associated with the outcome variable. Finally, variables with *p*-value < 0.05 were considered significantly associated with academic satisfaction. The Hosmer and Lemeshow goodness of fit test was used to check model fitness and indicated that the data was fitted for the model (*p* = 0.63).

### Ethics approval and consent to participants

Ethical clearance was obtained from Mekelle University College of health sciences, institutional board review (IBR) and a letter of cooperation was also received from the head department of midwifery, college of health sciences, Mekelle University to each Universities. The objective and the aim of the research was explained to the study subjects on the information sheet of the questionnaire, verbal and written informed consent was obtained from each study subject after explanation of the purpose of the study and involvement (to be a participant) was after their complete consent. Any health science students who were not willing to participate in the study were not forced to participate, no personal identifications were included in the data sheet and all data taken from the participants were kept strictly confidential and used only for the study purpose.

### Consent for publication

Not Applicable.

### Availability of data and material

The datasets generated and/or analyzed during the current study are not publicly available due to the personal computer of the corresponding author used to submit this manuscript corrupted the SPSS software and auto deleted the app from the computer but the datasets are putted in the hard disc as a backup and when the hard disc connected to the computer it does not read since the SPSS app is already deleted but the datasets are available from the corresponding author on reasonable request.

### Competing interests

The authors declare no any financial and non-financial competing interests.

### Duplicate publication

We confirm that this manuscript has not been published elsewhere and is not under consideration by another journal.

## Result

### Socio-demographic characteristics of participants

Out of the expected 850 participants, 850 gave a complete response with a response rate of 100% in both universities. From all participants of PBL and LBL three hundred eighty (89.4%)/ 378 (88.9%) were in the age group of 20–24 years, with a mean age of 20.8+ (1.1)/ 20.8+ [1] year and more than half 235(55.3%)/ 247 (58.1%) were male while 190 (44.7%)/ 178 (41.9%) were female participants. The majorities 314 (73.9%)/ 312 (73.4%) were Orthodox. Regarding ethnic group of participants 206 (48.5%)/ 63 (14.8%) of the respondents were Amhara. Out of the total respondents 122 (28.7%)/ 161 (37.9%) were from Medicine respectively. More than half 241 (56.7%) of the PBL participants and 139 (44.5%) LBL participants were second year while 184 (43.3%) of PBL and 236 (55.5%) of LBL were third year (Table 1).

**Table 1:** Socio-demographic characteristics of participants in selected Ethiopian Universities, Northern Ethiopia February 2021 (*n* = 850)

Background Characteristics	Number	
	PBL (n, %) *n* = 425	LBL (n, %) *n* = 425
Age		
15–19	45, (10.6)%	46, (10.8)%
20–24	380, (89.4)%	379, (89.2)%
Mean + SD	20.8 + (1.1)	20.8 + (1)
Sex		
Female	190, (44.7)%	178, (41.9)%
Male	235, (55.3)%	247, (58.1)%
Religion		
Orthodox	314, (73.9)%	312,(73.4)%
Muslim	46, (10.8)%	49,(11.5)%
Protestant	53, (12.5)%	62, (14.6)%
Other^a^	12, (2.8)%	2, (0.5)%
Ethnicity		
Amhara	206, (48.5)%	63, (14.8)%
Tigray	65, (15.3)%	269, (63.3)%
Oromo	84, (19.8)%	43, (10.1)%
Other^b^	70, (16.5)%	2, (0.5)%
Department		
Nurse	68,(16.0)%	62, (14.6)%
Medicine	122, (28.7)%	161, (37.90%
Midwifery	69, (16.2)%	45,(10.6)%
Pharmacy	57, (13.4)%	63,(14.8)%
C. laboratory	61, (14.4)%	57,(13.4)%
Anesthesia	48, (11.3)%	37,(8.7)%
Year of study		
Second year	241, (56.7)%	139, (44.5)%
Third year	184, (43.3)%	236, (55.5)%
Mother’s education status		
Cannot read and write	96, (22.6)%	92, (21.6)%
Can read and write	77, (18.1)%	93, (21.9)%
Elementary	48, (11.3)%	34, (8.0)%
Middle school	100,(23.5)%	76, (17.9)%
College/University	104, (24.5)%	130, (30.6)%
Father’ education status		
Cannot read and write	23, (5.4)%	23, (5.4)%
Can read and write	68, (16.0)%	73, (17.2)%
Elementary	69, (16.2)%	66, (15.5)%
Middle school	102, (24.0)%	95, (22.4)%
College/University	163, (38.4)%	168, (39.5)%

*C. laboratory* Clinical laboratory

N.B^a^ Catholic, Jobbah,

^b^ Guraghe, Somali, Afar, Sidama

### Personal factors of participants

Among 425 PBL participants, more than half (55.8%) and among 425 LBL participants 49.6% reported that their preferred learning style was matched with the teaching method that were applied in the University. Majorities (69.6%)/ (62.6%) of the respondents were deep learner whereas 30.4%/ 37.4% of the respondents were surface learner (Table 2).

**Table 2:** Percentage distribution of personal factors for academic satisfaction of participants in selected Ethiopian Universities, Northern Ethiopia February 2021

Variables	Number	
	PBL (n, %) ***n*** = 425	LBL (n, %) ***n*** = 425
**Preferred learning style**		
Yes	237, (55.8)%	211, (49.6)%
No	188, (44.2)%	214,(50.4)%
**CGPA**		
< 2.50	13, (3.1)%	24, (5.6)%
2.51–2.99	50,(11.8)%	88, (20.7)%
3.00–3.50	196,(46.1)%	189, (44.5)%
3.51–4.00	166, (39.1)%	124, (29.2)%
Mean + SD	3.35 + (0.4)	3.2 + (0.4)
**Classroom experience of distress**		
Yes	136, (32)%	177, (41.6)%
No	289,(68)%	248, (58.4)%
**Time investment for learning**		
Yes	285, (67.1)%	228, (53.6)%
No	140, (32.9)%	197, (46.4)%
**Energy and effort**		
Yes	159, (39.4)%	187, (44)%
No	266, (62.6)%	238, (56)%
**Approaches to learning**		
Deep learner	296, (69.6)%	266, (62.6)%
Surface learner	129, (30.4)%	159, (37.4)%
**Improvement in learning**		
Yes	321, (75.5)%	260, (61.2)%
No	104, (24.5)%	165, (38.8)%

### Institutional factors related to academic satisfaction of participants

Most PBL & LBL; 324 (76.2%)/ 307 (72.2%) respondents were happy with the quality of Instructors. More than half 64.9% of the PBL participants and 42.1% of LBL were satisfied with the curriculum appropriateness for their field of study (Table 3).

**Table 3:** Percentage distribution of Institutional factors for academic satisfaction of participants in selected Ethiopian Universities, Northern Ethiopia February 2021

Variables	Number	
	PBL (n, %) ***n*** = 425	LBL (n, %) ***n*** = 425
**Good quality of Instructors**		
Yes	324, (76.2)%	307, (72.2)%
No	101, (23.8)%	118,(27.8)%
**Curriculum appropriateness**		
Yes	276, (64.9)%	179, (42.1)%
No	149, (35.1)%	246, (57.9)%
**Understanding of learning objectives**		
Yes	321, (75.5)%	286, (67.3)%
No	104, (24.5)%	139, (32.7)%
**Helpfulness of the teaching method**		
Yes	295, (69.4)%	197, (46.4)%
No	130, (30.6)%	228, (53.6)%
**Student centeredness of the teaching learning process**		
Yes	302,(71.1)%	176, (41.4)%
No	123, (28.9)%	249,(58.6)%
**Timely and helpfulness of feedback given**		
Yes	298, (70.1)%	172, (40.5)%
No	127, (29.9)%	253, (59.5)%
**Presence of good quality of teaching**		
Yes	266, (62.6)%	241, (56.7)%
No	159, (37.4)%	184, (43.3)%
**Presence of good relation with Instructors**		
Yes	358, (84.2)%	340, (80)%
No	67, (15.8)%	85, (20)%
**Staff helpfulness**		
Yes	211, (49.6)%	196, (46.1)%
No	214, (50.4)%	229, (53.9)%
**Presence of good relation with classmates**		
Yes	388, (91.3)%	389, (91.5)%
No	37, (8.7)%	36, (8.5)%
**Acceptance of course contents**		
Yes	318, (74.8)%	282, (66.4)%
No	107, (25.2)%	143, (33.6)%
**Suitability of the learning environment**		
Yes	342, (80.5)%	338, (79.5)%
No	83, (19.5)%	87, (20.5)%
**Presence of good assessment method**		
Yes	284, (66.8)%	234, (55.1)%
No	141, (33.2)%	191, (44.9)%
**Fulfillment of classroom facilities**		
Yes	264, (62.1)%	215, (50.6)%
No	161, (37.9)%	210, (49.4)%
**Presence of facilities to support student’s learning**		
Yes	140, (32.9)%	142, (33.4)%
No	285, (67.1)%	283, (66.6)%
**Presence of adequate learning equipment**		
Yes	314, (73.9)%	246, (57.9)%
No	111, (26.1)%	179, (42.1)%
**Presence of adequate library material & facilities**		
Yes	301, (70.8)%	348, (81.9)%
No	124, (29.2)%	77, (18.1)%
**Accessibility of technology in the campus**		
Yes	227, (53.4)%	278, (65.4)%
No	198, (46.6)%	147, (34.6)%

### Academic satisfaction of participants

**Fig. 1:**
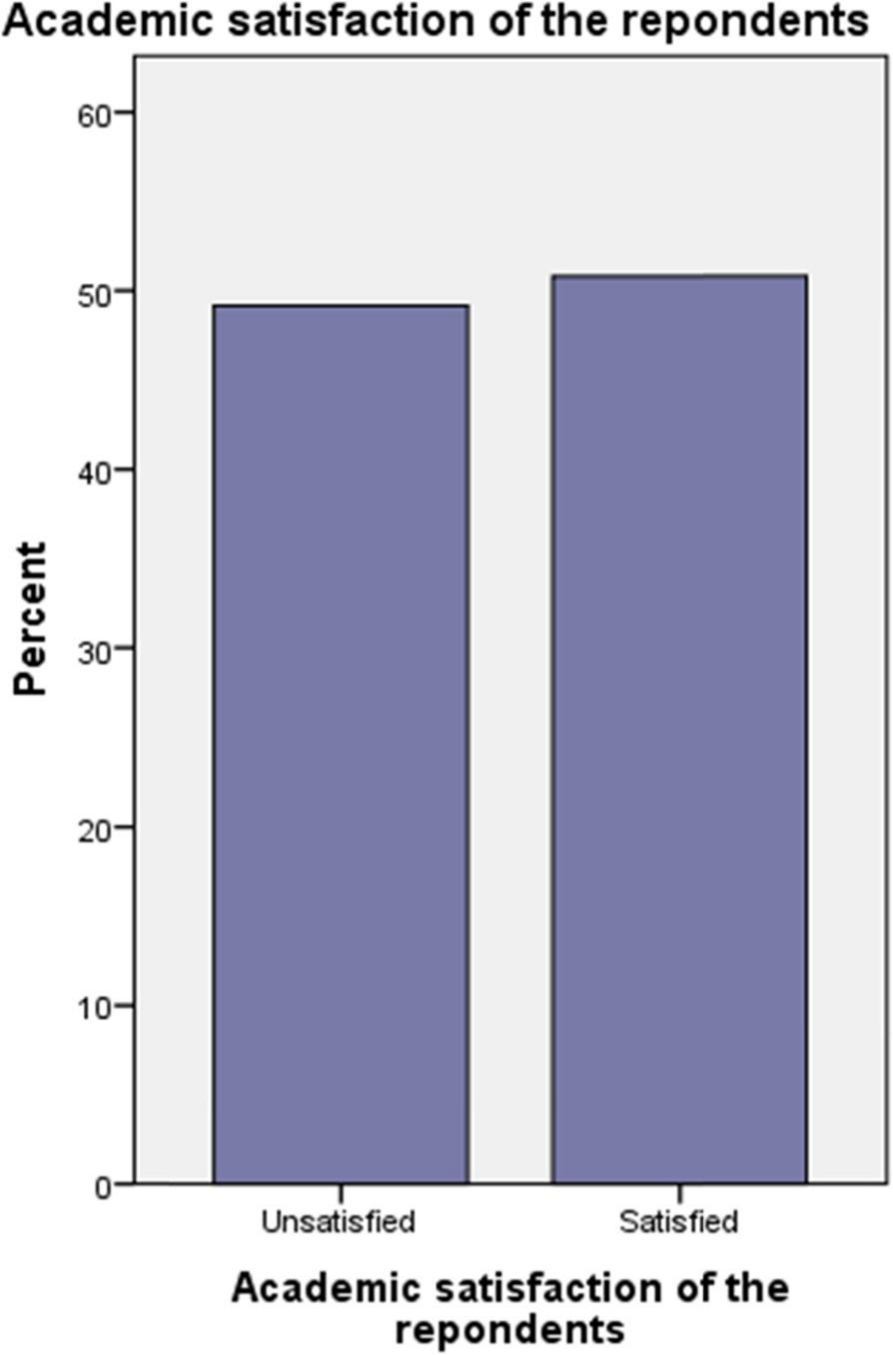
Magnitude of academic satisfaction on PBL among regular undergraduate health science University students, Northern Ethiopia, 2021

**Fig. 2:**
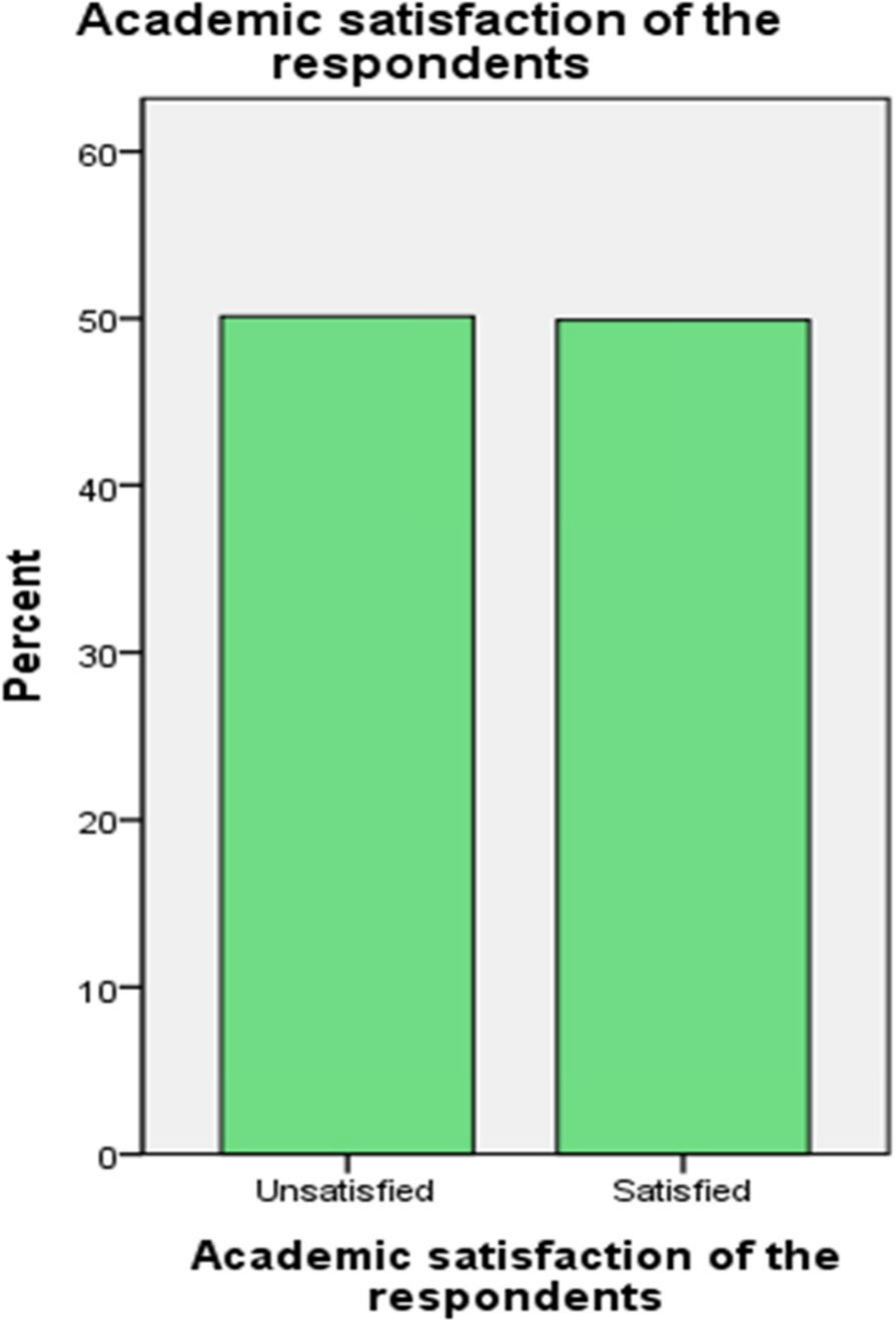
Magnitude of academic satisfaction on LBL among regular undergraduate health science University students, Northern Ethiopia, February 2021

The magnitude of academic satisfaction on PBL among the college of health science regular undergraduate University students in Ethiopia was 50.9% and LBL was 49.9%. And the satisfaction rate of PBL & LBL participants on academic activities were; teaching (64.7%/71.8%), learning (74.9%/67.5%), supervision & feedback (63.6%/39.6%), course organization (64.6%/57.9%), IT facility (46.1%/43.2%) & skill development (66.3%/44.7%) respectively **(**Figs. 1 and 2).

### Factors associated with academic satisfaction among PBL participants

In bi-variate logistic regression preferred learning style, experiences of distress in class room teaching learning process, appropriateness of respondent’s energy and effort used in their learning, improvement in learning, quality of teaching, type of curriculum implementing, relationship with classmates, acceptance of course contents, understanding of learning objectives, timely and helpfulness of feedback, assessment method, presence of facilities that support student’s learning, presence of adequate learning equipment, presence of adequate materials and facilities in the library and accessibility of technology were significantly associated; however, in multiple logistic regression type of curriculum implementing (AOR = 1.50, 95% CI = 1.02, 2.21), experience of classroom distress (AOR = 1.93, 95% CI = 1.22, 3.06), presence of quality of teaching (AOR = 0.54, 95% CI = 0.34, 0.86), presence of good relationship with classmates (AOR = 0.33, 95% CI = 0.13, 0.80), acceptance of course contents (AOR = 0.56, 95% CI = 0.33, 0.93) and accessibility of technology in the campus (AOR = 0.62, 95% CI = 0.40, 0.96) remain significantly associated with academic satisfaction (Table 4).

**Table 4:** Binary and Multiple logistic regression analysis of factors affecting for academic satisfaction among PBL students Northern Ethiopia, February 2021

Variables	Academic satisfaction		Odds ratio with 95% CI	
	Satisfied	Unsatisfied	COR	AOR
Type of curriculum implementing				
PBL	216	209	1.31 (1.05, 2.15) *	1.50 (1.02, 2.21)*
LBL	212	213	1.00^a^	1.00^a^
Preferred learning style of the respondents				
Yes	136	101	1.00^a^	
No	80	108	0.55 (0.37, 0.81)*	
Experience of respondents with distress in classroom teaching learning process				
Yes	52	84	1.00	1.00
No	164	125	2.11 (1.39, 3.21)*	1.93 (1.22, 3.06)*
Appropriateness of respondent’s energy and effort using in their learning				
Yes	91	68	1.00	
No	125	141	0.66 (0.44, 0.98)*	
Improvement of respondents in knowledge, skill &/ attitude				
Yes	176	145	1.00	
No	40	64	0.51 (0.32, 0.80)*	
Quality of teaching				
Yes	158	108	1.00	1.00
No	58	101	0.39 (0.26, 0.58)*	0.54 (0.34, 0.86)*
Respondent’s relationship with their classmates				
Yes	207	181	1.00	1.00
No	9	28	0.28 (0.12, 0.61)*	0.33 (0.13, 0.80)*
Respondent’s acceptance of course contents				
Yes	179	139	1.00	1.00
No	37	70	0.41 (0.26, 0.64)*	0.56 (0.33, 0.93)*
Respondent’s understanding of their learning objectives				
Yes	172	149	1.00	
No	44	60	0.63 (0.40, 0.99)*	
Timely and helpfulness of feedback				
Yes	164	134	1.00	
No	52	75	0.56 (0.37, 0.86)*	
Assessment method that Instructors are using				
Yes	160	124	1.00	
No	56	85	0.51 (0.33, 0.77)*	
Presence of facilities that support student’s learning				
Yes	83	57	1.00	
No	133	152	0.60 (0.39, 0.90)*	
Demonstration room fulfillment with adequate learning equipment				
Yes	171	143	1.00	
No	45	66	0.57 (0.36, 0.88)*	
Presence of adequate learning materials and facilities in the library				
Yes	165	136	1.00	
No	51	73	0.57 (0.37, 0.88)*	
Accessibility of technology to use in the campus				
Yes	133	94	1.00	1.00
No	83	115	0.51 (0.34, 0.75)*	0.62 (0.40, 0.96)*

Crude and adjusted = for preferred learning style, relationship with classmates, course content,

* **=** significant results

^a^ = Reference category

### Factors associated with academic satisfaction among LBL participants

Binary logistic regression analysis showed factors significantly associated with academic satisfaction among LBL students were ethnicities of the respondents, department, year of study, preferred learning style, experiences of classroom distress, appropriateness of energy and effort, improvement in learning, presence of quality of teaching, staff helpfulness, presence of good relationship with classmates, curriculum appropriateness, acceptance of course contents, timely and helpfulness of feedback, fulfillment of classroom facilities, presence of facilities that support student’s learning, presence of adequate learning equipment, presence of adequate learning materials in the library and accessibility of technology in the campus with a *P*- value of less than 0.05; however, in multiple logistic regression year of study (AOR = 0.29, 95% CI = 0.17, 0.48), appropriateness of energy and effort used in their learning (AOR = 0.55, 95% CI = 0.35, 0.88), presence of quality of teaching (AOR = 0.51, 95% CI = 0.31, 0.85) and acceptance of course contents (AOR = 0.59, 95% CI = 0.35, 0.97) remain significantly associated with the outcome variable (Table 5).

**Table 5:** Binary and Multiple logistic regression analysis of factors affecting for academic satisfaction among LBL students Northern Ethiopia, February 2021

Variables	Academic satisfaction		Odds ratio with 95% CI	
	Satisfied	Unsatisfied	COR	AOR
Ethnicity of the respondents				
Amhara	38	25	0.53 (0.23, 1.23)	
Tigray	121	148	0.81 (0.36, 1.82)	
Oromo	28	15	0.43 (0.22, 0.85)*	
Others	25	25	1.00^a^	
Department of the respondents				
Nurse	26	36	1.00	
Medicine	84	77	0.68 (0.30, 1.55)	
Pharmacy	31	32	1.03 (0.50, 2.11)	
Midwifery	31	14	0.91 (0.40, 2.06)	
C. laboratory	21	36	2.09 (0.85, 5.17)	
Anesthesia	19	18	0.55 (0.23, 1.28)*	
Year of study of the respondents				
Second year	75	114	0.47 (0.32, 0.70)*	0.29 (0.17, 0.48)*
Third year	137	99	1.00	1.00
Preferred learning style of the respondents				
Yes	119	92	1.00	
No	93	121	0.59 (0.40, 0.87)*	
Experience of respondents with distress in classroom teaching learning process				
Yes	77	100	1.00	
No	135	113	1.55 (1.05, 2.28)*	
Appropriateness of respondent’s energy and effort using in their learning				
Yes	106	81	1.00	1.00
No	106	132	0.61 (0.41, 0.90)*	0.55 (0.35, 0.88)*
Improvement of respondents in knowledge, skill &/ attitude				
Yes	140	120	1.00	
No	72	93	0.66 (0.44, 0.98)*	
Quality of teaching				
Yes	140	101	1.00	1.00
No	72	112	0.46 (0.31, 0.68)*	0.51 (0.31, 0.85)*
Respondent’s idea regarding staff helpfulness				
Yes	111	85	1.00	
No	101	128	0.60 (0.41, 0.88)*	
Respondent’s relationship with their classmates				
Yes	201	188	1.00	
No	11	25	0.41 (0.19, 0.86)*	
Curriculum appropriateness for field of study				
Yes	102	77	1.00	
No	110	136	0.61 (0.41, 0.90)*	
Respondent’s acceptance of course contents				
Yes	156	126	1.00	1.00
No	56	87	0.52 (0.34, 0.78)*	**0.59 (0.35, 0.97)** *
Timely and helpfulness of feedback				
Yes	97	75	1.00	
No	115	138	0.64 (0.43, 0.95)*	
Fulfillment of classroom facilities				
Yes	119	96	1.00	
No	93	117	0.64 (0.43, 0.94)*	
Presence of facilities that support student’s learning				
Yes	84	58	1.00	
No	128	155	0.57 (0.37, 0.85)*	
Demonstration room fulfillment with adequate learning equipment				
Yes	138	108	1.00	
No	74	105	0.55 (0.37, 0.81)*	
Presence of adequate learning materials and facilities in the library				
Yes	188	160	1.00	
No	24	53	0.38 (0.22, 0.65)*	
Accessibility of technology to use in the campus				
Yes	151	127	1.00	
No	61	86	0.59 (0.39, 0.89)*	

Crude and adjusted = for ethnicity, department, year of study, preferred learning style, experience of classroom distress, classroom facilities, learning equipment and library facilities

*= significant results

^a^ *=* Reference category

## Discussion

This Institution based comparative cross sectional study tried to assess and compare academic satisfaction and identified factors affecting the academic satisfaction of regular undergraduate health science students in selected Universities with different instructional methods.

In this study, it was found that the magnitude of academic satisfaction of regular undergraduate health science students on PBL was 50.9% and their counterpart was about 48.9%. This study finding is not consistent with the study conducted in Turkey University which showed that the satisfaction of undergraduate medical students with PBL instructional method was 64.6%. The possible reason might be due to the difference in the study participants which the current study included most of the departments under health science unlike the study conducted in Turkey University which were involved medical students exclusively and study setting [16].

This study revealed that factors associated with the academic satisfaction of PBL students as experience of distress in classroom, quality of teaching, relationship with classmates, course content and accessibility of technology to use in the campus where as factors significantly associated with the satisfaction of LBL students were year of study, energy and effort, quality of teaching and course content. In general factors that affect the satisfaction of PBL and LBL students in common were type of curriculum, quality of teaching and course content.

In this survey PBL students were more likely to be academically satisfied than LBL students with the type of curriculum implementing in their campus. Since, there is no similar research done for the factors of academic satisfaction with the adjusted odds ratio the authors could not compare this research finding with the previous findings in this discussion part.

The result of this study revealed that PBL students who had not experienced distress in classroom teaching learning process were two times more likely to be academically satisfied than students who had experience. As per the authors search since, there is no similar research done for the factors of academic satisfaction with the adjusted odds ratio could not compare this research finding with the previous findings in the discussion part.

Quality of teaching is another factor that affects the academic satisfaction of University students [17]. According to this survey finding PBL students who were not satisfied with the quality of teaching were less likely to be academically satisfied than the students who were satisfied with the quality of teaching in their campus.

Similarly, LBL students who were not satisfied with the quality of teaching were also less likely to be academically satisfied than the students who were satisfied with quality of teaching in the campus. Since, there is no similar research done for the factors of academic satisfaction with the adjusted odds ratio could not compare this research finding with the previous findings in this discussion part.

Problem based learning students who were not satisfied with the relationship they had with their classmates were less likely to be academically satisfied than their counterparts.

Both PBL and LBL participants who were not satisfied with the course contents of their main subjects were less likely to be academically satisfied than their counterparts.

Accessibility of technology is also one major factor contributing for student’s satisfaction in higher education institutions. As of this study result PBL students who were not satisfied with accessibility of technology to use in the campus were less likely to be academically satisfied than those who were satisfied with. This might be due to the fact that PBL instructional method by its nature is mostly dependent on accessibility of technology that all students should get access to the internet even in the PBL sessions to make student’s learning process evidence based. Similar reason here as the above not to compare this study finding with others.

This study showed that year of study of the respondents among LBL had a significant influence on the academic satisfaction of students. Being a second year student is less likely to be academically satisfied than being a third year.

According to this research finding Lecture based learning students who were not satisfied with the appropriateness of energy and effort using in their learning were less likely to be academically satisfied than their counterpart. In order to compare this finding with others finding the authors could not get similar research findings.

### Limitation of the study

- This study did not include students other than regular, which might affect its generalizability.
- In general, as per the authors search the articles that we found in this area were descriptive in nature and we could not find literatures done using logistic regression analysis with AOR similar with this study finding so we could not compare our study result with other researcher’s finding in the discussion part.

## Conclusion

The magnitude of academic satisfaction among PBL students was higher than LBL students in selected higher education institutions of Ethiopia as this study result showed. Experience of distress in classroom, quality of teaching, relationship with classmates, course content and accessibility of technology to use in the campus were statically associated with the outcome variable independently among problem based learning participants were as year of study, energy and effort, quality of teaching and course content were factors that are statically associated with the outcome variable independently among lecture based learning participants.

This research revealed that the magnitude of academic satisfaction among PBL students was higher than LBL students. Ministry of science and higher education and educational experts may give attention to improve the academic satisfaction of students learning with LBL method to ensure quality education across all the universities to produce qualified health care providers there by decrease the health issues of the community. This can be address by incorporating PBL across all higher education institutions as a formal instructional method and making use of appropriately to make health science students satisfied with the academic activities in their campus and make them competent in their profession.

### Recommendation

#### To ministry of science & higher education

Incorporating PBL as a formal instructional method in the curriculum across higher education institutions of Ethiopia to enhance the academic satisfaction of health science students thereby increase and maintain the quality of education and achieve one of its missions.

Since energy and effort that the LBL respondents are investing in their learning is one influencing factor of academic satisfaction in this study incorporating PBL method will resolve the problem as in its nature is constructivist that the students are the owner of their learning and involve in the construction of their own understanding to the subject matter and developing this habit will help them to use appropriate energy and effort in their learning process.

#### To the university administrators

In order to improve the academic satisfaction of PBL students the University administrators of DTU need to make sure that the accessibility of technology in the campus.

It is necessary that to assess and check the quality of education periodically in both universities by preparing quality measurement tools of the teaching learning process even by participating students. In addition it’s better for administrators to prepare and facilitate programs that aim in curriculum review to make it acceptable to the students of interest.

#### To researchers

It is recommended that researchers are better to do more research in this area by including all students of higher education institutions regardless of their academic program in health science to improve the quality of health science students there by increasing the quality of health services given in the country.

Further study of factors associated with the academic satisfaction of higher education institutions using the regression analysis is recommended.

## Data Availability

The datasets generated and/or analyzed during the current study are not publicly available due to the personal computer of the corresponding author used to submit this manuscript corrupted the SPSS software and auto deleted the app from the computer but the datasets are putted in the hard disc as a backup and when the hard disc connected to the computer it does not read since the SPSS app is already deleted but the datasets are available from the corresponding author on reasonable request.

## Abbreviations

AOR: Adjusted odds ratio
CI: Confidence interval
COR: Crude odds ratio
DTU: Debretabor University
IT: Information Technology
LBL: Lecture Based Learning
MU: Mekelle University
PBL: Problem Based Learning
SPSS: Statistical Package for Social Science
SRS: Simple Random Sampling
TL: Traditional Learning
